# HIV and diabetes impair *M. tuberculosis*-specific interferon-gamma responses on QuantiFERON-TB Gold Plus testing

**DOI:** 10.5588/ijtldopen.24.0011

**Published:** 2024-05-01

**Authors:** J.S. Zifodya, J.S. Kreniske, T.M. Temu, S.J. Masyuko, E.F. Attia, J.N. Mogaka, D. Onyango, S.T. Page, K. Crothers, J. Kinuthia, C. Farquhar, S.M. LaCourse

**Affiliations:** ^1^Section of Pulmonary, Critical Care, & Environmental Medicine, Tulane University School of Medicine, New Orleans, LA,; ^2^Division of Pulmonary and Critical Care Medicine, Weill Cornell Medicine, New York, NY,; ^3^Department of Global Health,; ^4^Division of Pulmonary, Critical Care, & Sleep Medicine, and; ^5^School of Nursing, University of Washington, Seattle, WA, USA;; ^6^Kisumu County Department of Health, Kisumu, Kenya;; ^7^Division of Metabolism, Endocrinology and Nutrition, University of Washington, Seattle, WA,; ^8^Veterans Affairs Puget Sound Healthcare System, Seattle, WA, USA;; ^9^Department of Research & Programs, Kenyatta National Hospital, Nairobi, Kenya;; ^10^Department of Medicine, Division of Allergy & Infectious Diseases, and; ^11^Department of Epidemiology, University of Washington, Seattle, WA, USA

**Keywords:** latent TB, interferon-gamma, HIV, DM, QFT-Plus

Dear Editor,

TB is the leading cause of death among people with HIV (PWH).^1^ One-quarter of the global population is infected with TB (TBI), of whom 5–10% will develop active TB, a risk increased 20-fold by HIV co-infection.^1,2^ Interferon-gamma (IFN-γ) release assays (IGRAs) are the most predictive tests for TBI progression.^3^ However, these assays have consistently demonstrated reduced sensitivity among PWH.^4–6^ The QuantiFERON-Plus (QFT-Plus) IGRA, which measures IFN-γ production to TB antigen stimulation in CD4+ and CD4+/CD8+ specific tubes, was developed to improve sensitivity including among PWH.^7^ We evaluated IFN-γ response to TB antigen stimulation measured by QFT-Plus among Kenyan adults with and without HIV. We hypothesized PWH would have reduced Mtb-specific IFN-γ responses to QFT-Plus and that CD4+ count and viral load would negatively influence IFN-γ response.

This is a cross-sectional sub-study in a previously described cohort of adults evaluated for cardiovascular and pulmonary disease in Kisumu, Kenya.^8^ Blood was collected for QFT-Plus testing for *Mycobacterium tuberculosis* (MTb) between December 2018 to December 2019. We enrolled a convenience sample of PWH (≥30-years old) from an HIV clinic and sex-matched people without HIV (PWoH) from HIV testing centers. PWH were on antiretroviral therapy (ART) for at least 6 months. Exclusion criteria included pregnancy and self-reported acute respiratory infections (including active TB symptoms). Questionnaires were performed at enrollment. Diabetes mellitus (DM) diagnosis was self-reported. For PWH, HIV RNA viral load (VL) and CD4 counts were obtained in the parent study or abstracted from medical records. Prior TB diagnosis and isoniazid prevention therapy (IPT) were self-reported and confirmed by chart review where possible. QFT-Plus testing was performed at enrollment per the manufacturer’s (Qiagen; Hilden, Germany) protocol.^7^ The QFT-Plus measures IFN-γ response (IU/ml) in a nil tube (negative control, measuring endogenous IFN-γ), a mitogen tube (positive control, measuring IFN-γ response to a non-specific T-cell stimulator), and two TB-specific tubes (TB1 and TB2) which elicit CD4+ and CD8+ T-cell responses to Mtb-specific antigens. The test is positive if the TB1 and/or TB2 IFN-γ response minus nil is ≥0.35 IU/ml. Indeterminate results are due to high nil or low mitogen response.^7^ We defined borderline results as 0.20–0.70 IU/ml.^9^ We compared prevalence of TBI (positive QFT-Plus), absolute IFN-γ levels (TB1 and TB2 minus nil), borderline, and indeterminate results between PWH and PWoH. Wilcoxon rank-sum test was used to compare medians. Univariate and multivariable linear regression analyses were used to evaluate factors (selected a priori) associated with quantitative TB-specific IFN-γ response. Ethical approval was obtained from the University of Washington Institutional Review Board and the Kenyatta National Hospital/University of Nairobi Ethical and Scientific Review Committee. Written informed consent was obtained from all study participants.

We enrolled 398 participants: 221 (56%) were PWH, 201 (51%) were women, and 14 (4%) had DM. Median age in PWH and PWoH was 47 (interquartile range [IQR] 41‒55) and 46 (IQR 34‒59) years respectively (*P* = 0.05). PWH had a median 10.2 years since HIV diagnosis, median CD4 count of 510 cells/mm^3^ (IQR 660‒360), and 20% had detectable VL (>20 copies/mL). Sixty (27%) PWH reported prior TB compared to 7 (4%) PWoH (*P* < 0.001). Five (2.3%) PWH and nine (5.1%) PWoH had DM (*P* = 0.21). Sex distribution was similar across groups. Among PWH, 178 (81%) reported prior IPT and none among PWoH. Overall, 46% of PWH and 61% of PWoH had a positive QFT-Plus test (*P* = 0.003). IFN-γ responses to mitogen and nil were lower among PWH compared to PwoH (*P* = 0.002 and *P* < 0.0001, respectively). Among QFT-Plus positive individuals, TB1- and TB2-specific IFN-γ levels were similar in PWH and PwoH (*P* = 0.09, *P* = 0.33). Three (1.4%) PWH and 6 (3.3%) PwoH had indeterminate results (*P* = 0.20). Thirty-one (14%) PWH and 28 (16%) PwoH had borderline MTb (TB1 or TB2) IFN-γ results (*P* = 0.72). We tested the sensitivity of a cut-off value of 0.2 IU/ml for PWH and CD4 <200 cells/mm^3^; among 18 participants with HIV and CD4 <200 cells/mm^3^, 50% had positive QFT-plus by standard cut-offs. Reduction in cut-off value to 0.20 IU/ml did not result in any further positive tests. In multivariable regression analyses (see Table; univariable analyses presented in Supplementary Data), HIV was significantly associated with diminished IFN-γ response in both TB1 and TB2 testing (*P* = 0.0003, *P* = 0.002). Age was associated with reduced Mtb IFN-γ responses among PWoH (TB1: *P* = 0.003; TB2: *P* = 0.008). Similarly, DM was associated with lower IFN-γ responses (TB1: *P* = 0.04; TB2: *P* = 0.03), although not in PWH. Among PWH, lower CD4 counts were associated with diminished responses (TB1: *P* = 0.03; TB2: *P* = 0.03). Self-reported prior IPT was not associated with IFN-γ response. In sensitivity analyses limited to those without prior IPT (including 43 PWH), the relationship of HIV and lower IFN-γ response was attenuated in all participants (TB1: *P* = 0.07; TB2: *P* = 0.14), and similar among QFT-Plus positive participants (TB1: *P* = 0.12; TB2: *P* = 0.25).

**Table.** Multivariable* analyses of factors associated with Mtb-specific interferon-γ response levels.

	Entire cohort TB1-nil (95% CI)	*P*-value	Entire cohort TB2-nil (95% CI)	*P*-value	PWH TB1-nil (95% CI)	*P*-value	PWH TB2-nil (95% CI)	*P*-value
HIV	–1.1 (–1.7 to –0.52)	0.0003	–0.97 (–1.6 to –0.37)	0.002	—	—	—	—
Age, years	–0.03 (–0.06 to –0.01)	0.003	–0.03 (–0.05 to –0.01)	0.008	–0.04 (–0.08 to 0.001)	0.05	–0.03 (–0.07 to 0.01)	0.14
Diabetes	–1.57 (–3.1 to –0.04)	0.04	–1.7 (–3.3 to –0.18)	0.03	–1.98 (–4.83 to 0.87)	0.17	–2.13 (–5.11 to 0.85)	0.16
Prior TB	0.02 (–0.76 to 0.82)	0.95	–0.20 (–1.0 to 0.59)	0.62	0.23 (–0.62 to 1.08)	0.61	–0.05 (–0.94 to 0.85)	0.92
Female sex	0.24 (–0.33 to 0.80)	0.41	0.23 (–0.34 to 0.81)	0.43	0.57 (–0.21 to 1.35)	0.15	0.51 (–0.31 to 1.32)	0.22
CD4+ T-cell count^†^	—	—	—	—	0.19 (0.02 to 0.37)	0.03	0.21 (0.03 to 0.39)	0.03
Detectable viral load^‡^	—	—	—	—	–0.53 (–1.47 to 0.41)	0.27	–0.51 (–1.49 to 0.47)	0.31
IPT^§^	—	—	—	—	–0.60 (–1.58 to 0.38)	0.23	–0.63 (–1.65 to 0.39)	0.23

* Linear regression of cofactors associated with TB1 or TB2 minus nil; adjusting for listed covariates; limited to participants with valid QFT-Plus tests, excluded 3 PWH (2 high nil and 1 low mitogen) and 6 people without HIV (all high nil).

^†^ Per 100 cells/mm^3^ CD4+ T-cell count increase.

^‡^ Detectable viral load as a yes/no variable, detectable is >20 copies/mL.

^§^ In sensitivity analyses, we included IPT in analyses for the entire cohort and it was not significantly associated with interferon-γ response.

Mtb = *Mycobacterium tuberculosis*; CI = confidence interval; PWH = people with HIV; IPT = isoniazid prevention therapy.

Our analyses demonstrate diminished Mtb-specific IFN-γ response among PWH using QFT-Plus testing. Lower CD4 count was associated with lower IFN-γ response to both TB antigens. Overall test positivity was lower among PWH, suggesting reduced sensitivity. In this cross-sectional study we cannot definitively establish this difference in sensitivity. TBI prevalence was high among PWoH; this high prevalence was similar to prior surveys among healthcare workers and school workers in Kisumu.^10^ Prior IPT use did not modulate IFN-γ response among PWH. For participants with CD4<200 cells/mm^3^, we did not find increased test sensitivity with a lower cut-off value at 0.2 IU/ml, however analyses were limited by the small number of participants with severe immunosuppression (*n* = 18). The present study supports the hypothesis that among individuals with controlled HIV on ART, HIV infection is associated with diminished Mtb-specific IFN-γ response with QFT-Plus testing, as with prior iterations of IGRA, and greater immunosuppression is associated with further reduction in this response. Interestingly, we also found lower IFN-γ response among PWoH with DM. Interestingly, this effect was not observed among PWH, potentially due to the already muted IFN-γ response associated with positive HIV status and the small number of participants with DM in this cohort. Conflicting reports exist concerning the influence of DM on IGRA testing, with some analyses suggesting increased IFN-γ response.^11–13^ The large proportion of participants with borderline results reiterate previous concerns on test specificity.^9,14^ We did not evaluate immunosuppression other than with HIV and DM. Further study is needed to determine the optimal operating parameters for IGRA testing among patients with HIV, DM, and other immunologically significant comorbidities.

Our analyses demonstrate substantially diminished IFN-γ response among PWH in response to QFT-Plus testing, particularly those with lower CD4 counts, as well as PWoH with DM. Additionally, many participants had borderline results. Our study brings into question the appropriateness of current cut-offs for the QFT-Plus, particularly in PWH and those with comorbidities impacting immune response. These findings suggest that TBI is likely underdiagnosed in PWH and people with DM despite these groups being most at risk for progression to active TB disease. Therefore, further research is urgently needed to identify optimally predictive cut-off values for Mtb-specific IFN-γ response in TBI testing for these vulnerable populations.
